# Effects of heat stress on performance, physiological parameters, and blood profiles of early-fattening Hanwoo steers in climate chambers

**DOI:** 10.5713/ab.23.0274

**Published:** 2023-10-20

**Authors:** Jun Sik Woo, Na Kyun Lee, Hong Gu Lee, Keun Kyu Park

**Affiliations:** 1 Department of Animal Science and Technology, Konkuk University, Seoul 05029, Korea

**Keywords:** Fattening Period, Hanwoo Steers, Heat Stress, Heart Rate, Rectal Temperature, Temperature Humidity Index

## Abstract

**Objective:**

This study was conducted to assess effects of heat stress on growth performance, physiological parameters, and blood profiles of Hanwoo steers during early-fattening period in climate chambers.

**Methods:**

Four Hanwoo steers (body weight, 454.3±10.9 kg; age, 14±0.1 month) were allocated into four levels of temperature-humidity index (THI) in a 4×4 Latin square design for 21 days (pre-adaptation, 7 d; heat stress, 7 d; post-adaptation, 7 d) per period. Experimental treatments were assigned according to THI chart based on National Institute Animal Science (NIAS, 2022): Comfort (25.5°C to 26.5°C, 60%; THI 73 to 75), Mild (28°C to 29°C, 60%; THI 77 to 79), Moderate (29.5°C to 30.5°C, 80%; THI 82 to 84), and Severe (31°C to 32°C, 80%; THI 85 to 86) in separate climatic controlled chambers.

**Results:**

The dry matter intake (DMI) of the formula feed was lower in Severe compared to Mild and Comfort (p<0.05). The DMI of rice straw was the lowest in Severe and lower in Moderate than Comfort and Mild (p<0.05). Both average daily gain and feed conversion ratio of Severe and Moderate were lower than those of Mild and Comfort (p<0.05). Water intake was the highest in Severe and lower in Moderate compared with Comfort and Mild (p<0.05). Heart rate and rectal temperature increased as THI level increased (p<0.05). Glucose was the lowest in Severe and lower in Moderate compared to Comfort (p<0.05). On the contrary, non-esterified fatty acid was the highest in Severe and lower in Moderate compared with Comfort (p<0.05). Blood urea nitrogen of Moderate and Severe were higher than those of Comfort and Mild (p<0.05). Cortisol increased as THI increased (p<0.05).

**Conclusion:**

This study demonstrated the negative effects of heat stress on the performance and physiological responses of Hanwoo steers during the early-fattening period. In addition, it is judged that the THI chart for Hanwoo steers of National Institute of Animal Science (2022) was properly calculated.

## INTRODUCTION

The Fifth Assessment Report of Intergovernmental Panel on Climate Change (IPCC) indicated the range of increase in global average surface temperature will be between 0.3°C and 4.8°C by 2100 [1]. These potential risks are deeply associated with animal productivity by environmental parameters such as heat stress [2,3]. Ruminants exposed to heat stress by severe climatic changes will have negatively affected growth, productivity, reproduction and welfare [4], and physiological and energy metabolic changes will occur for maintaining their own normal body temperature [5]. Economic losses caused by high temperature stress are estimated to be approximately $1 billion for dairy and $370 million for beef industries in the USA [6].

To monitor and reduce such losses, temperature-humidity index (THI) has been promoted as an indicator of identifying heat stress in both dairy and beef cattle [7–9]. The THI is a value obtained by combining the factors of air temperature and humidity to reflect the level of thermal stress. The THI chart is typically represented on a scale of 0 to 100, where higher values indicate a greater degree of heat stress. This chart conveniently facilitates the assessment of heat stress levels, enabling easy identification of the extent to which animals may be affected. It proves particularly valuable in the prediction and management of heat stress, which can have implications for the health and productivity of livestock.

Many studies have been conducted on heat stress in various types of beef and dairy cattle [9,10], but data on Hanwoo (*Bos taurus coreanae*), a Korean native beef cattle, are limited. Some studies have been focused on the effects according to levels of THI in Hanwoo calves in climatic controlled chambers [11–15], and nutritional effects of various feed ingredients in Hanwoo steers on summer feedlot [16,17]. Although the National Institute Animal Science of Korea (NIAS [18]) provided the THI chart for Hanwoo, the chart was based on very limited data. Therefore, a more precise assessment is required to ensure an accurate evaluation.

In addition, in the case of Hanwoo steers, the period from 13 to 21 months of age is after passing the growing period and when feed intake increases to its maximum according to the development of their rumen. This leads to compensatory growth instead of restricted feeding during the growing period, resulting in an increase not only in muscle growth but also in the accumulation of intramuscular fat and body fat. However, excessive feeding during the early-fattening period can cause side effects such as thickening of subcutaneous fat and significant accumulation of non-digestible fat (i.e., perirenal fat), so it is important to limit the feeding of formula feed with appropriate levels of energy and crude protein (CP). Severe heat stress during this period could lead to a decrease in feed intake and subsequent reduction in productivity, consequently adverse effects on the intramuscular fat level and meat quality grade in the late stage of fattening [19].

For precise measurement of the relationship between metabolic changes of Hanwoo steers and their environment, chamber experiments under strictly controlled climate conditions, rather than just outdoor conditions, are vital. This is because there are many variables in the outdoor environment such as insolation, airflow, and precipitation. Therefore, the aim of this study was to assess the effects of level of heat stress on physiological parameters, blood profiles, and growth performance of Hanwoo steers during the early-fattening period in climatic controlled chambers.

## MATERIALS AND METHODS

### Animal care

The experimental protocol was reviewed and approved by the Institutional Animal Care and Use Committee at Konkuk University (Approval number: KU21088).

### Chemical analysis

All samples of diets were dried at a forced-air oven for over 24 hours at 105°C and ground through a 2 mm screen using a Wiley mill (Model 4; Thomas Scientific, Swedesboro, NJ, USA). The diets were analyzed for dry matter (DM; method 930.15), CP (method 976.05), ether extract (EE; method 2003.05), ash (method 942.05), neutral detergent-insoluble fiber (NDF; method 2002.04), acid detergent-insoluble fiber (ADF), and lignin (method 973.18) as described in AOAC [20].

### Animal trial

**Figure 1:**
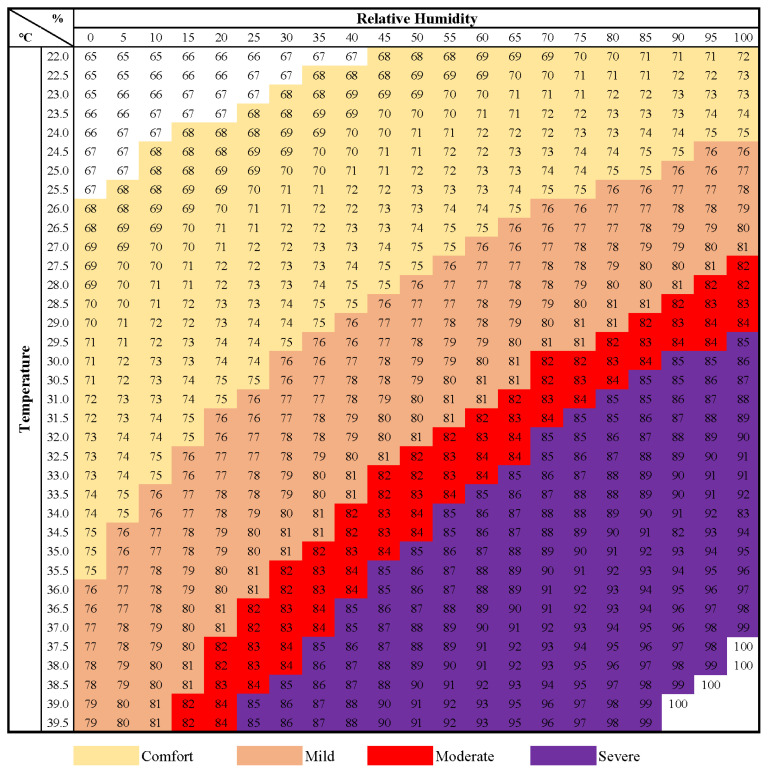
Temperature-humidity index by heat stress level in fattening stage of Hanwoo steers (adopted from [18]).

Four early-fattening Hanwoo steers (body weight [BW] 454.3 ±10.9 kg; age, 14±0.1 month) were allocated into four levels of THI in a 4×4 Latin Square design using a spreadsheet program developed by Kim and Stein [21]. The goal of this experiment was to determine the performance and physiological responses of individuals in an environmentally controlled chamber. Due to the high individual variation in large frame ruminants, it was determined that Latin Square design would help increase the number of experimental units under conditions of small number of animals and improve the accuracy of the experiment. Each experimental period was conducted for 21 days, having 7 days of pre-adaptation, heat stress, and post-adaptation, respectively. Treatments were assigned according to THI chart: Comfort (25.5°C to 26.5°C, 60%; THI 73 to 75), Mild (28°C to 29°C, 60%; THI 77 to 79), Moderate (29.5°C to 30.5°C, 80%; THI 82 to 84), and Severe (31°C to 32°C, 80%; THI 85 to 86) based on NIAS (Figure 1) [18]. The THI was calculated using the equation ([1.8×T_db_+32]–[0.55–0.0055×RH]×[1.8×T_db_–26]; T_db_, dry bulb temperature; RH, relative humidity) by National Research Council (NRC) [8].

Each steer was assigned to one of four separate climatic controlled chambers, measuring 3.5×3×3 m (length×width× height). Ambient temperature and RH of the chambers were regulated from 0800 to 1800 h according to the treatments. During the nighttime between 1800 and 0800 h, THI was kept below 76, assuming that the value did not affect the physiological parameters of animals [22]. This was done to simulate natural conditions in which temperatures drop during the night to alleviate heat stress and prevent animal mortality, particularly in severe THI conditions. Steers were acclimatized to the chambers at Comfort level for pre-adaptation days per each experimental period. After heat stress, all animals left the chambers and were rested in an individual pen (5×10 m) during the post-adaptation days. Climatic variables in each chamber were compiled by using an ambient temperature and RH logger (MHT-381SD; Lutron Electronics Inc., Coopersburg, PA, USA). Experimental diets were commercial early-fattening stage formula feed and rice straw. The chemical composition of experimental formula feed and rice straw are displayed in Table 1. The formula feed were approximately offered 1.8% (as-fed basis) of BW per steer and the ratio of roughage to formula feed was fixed at 30:70 as suggested by NIAS [19].

Feeds were offered twice equally at 0900 and 1700 h daily. Fresh water and mineral blocks were provided without constraint during the experimental period. Feed and water residuals were recorded before the next morning feeding. Individual BW was obtained twice per period at chamber-in and -out. The dry matter intake (DMI) was measured every period for calculating feed conversion ratio (FCR; feed/gain).

### Physiological parameters

Heart rate (HR) was determined using a stethoscope by measuring beat per minute (bpm) of the heart. Rectal temperature (RT) was measured after blood collection using a digital thermometer (KD-133; Polygreen Co., Ltd., Berlin, Germany). To accurately assess RT of steers, the thermometer was inserted 3 cm deep into the rectum of steers and maintained in contact with the mucosa for a duration of one minute [15]. The HR, RT and blood were collected on 0, 3, and 7 d of the heat stress phase per period at 1400 h, assuming the time of day when heat stress is at its peak.

### Blood profiles

Blood samples were obtained from jugular venipuncture to both non-heparinized vacutainers (10 mL; Becton-Dickinson, Franklin Lakes, NJ, USA) and ethylenediaminetetraacetic acid-treated vacutainers (10 mL; Becton-Dickinson, USA). Serum samples were separated using a centrifuge at 2,700 g at 4°C for 15 minutes and transferred to a 1.5 mL tube (Eppendorf AG, Hamburg, Germany) for storing at −80°C until analysis.

Serum samples for analyzing glucose, triglyceride, albumin, blood urea nitrogen (BUN), total protein, high-density lipoprotein, and total cholesterol were used to an automated chemistry analyzer (CHEM 7000i; Fujifilm, Tokyo, Japan). Serum cortisol was analyzed using a Bovine ELISA test kit (MBS70325; MyBioSource Inc., San Diego, CA, USA), and an analytical reagent for measuring non-esterified fatty acid (NEFA) was obtained from WAKO (Osaka, Japan). Whole blood samples (i.e. lymphocytes) were assessed by using a VetScan HM2 analyzer (Abaxix Inc., Union City, CA, USA).

### Calculations and statistical analysis

Total digestible nutrient (TDN) and FCR value of experimental feeds were calculated as follows:

- TDN = 0.93×CP+0.92×(1+EE–ash–CP–NDF)+0.75 ×(NDF– ADL)×(1– ADL2/3/(NDF)2/3) [23].
- FCR = DMI (kg)/BW gain (kg).

All experimental data were analyzed using the MIXED procedure of SAS (SAS Inst. Inc., Cary, NC, USA). The statistical model included that treatments were treated as fixed effects, and animals and periods were treated as random effects. Least squares means of each treatment were calculated, and compared using the PDIFF option when the treatment effect was significant. Statistical significance was declared at an alpha level of less than 0.05.

## RESULTS AND DISCUSSION

### Animal performance

In order to finely determine the effects of relationship between THI changes and animals, it is essential to conduct experiments in a climatic controlled chamber. Particularly, because ruminants exhibit high inter-individual variability, repeated measurements under varying environmental conditions of THI using ruminants of the same age and similar body weight can help to enhance the accuracy of the experiment. Therefore, the Latin Square design can effectively reduce or control experimental errors with a small number of experimental units. In other word, it can both minimize error and enhance accuracy in the experiment by increasing the sensitivity through a two-way blocking of animals and periods.

The DMI, BW gain, average daily gain (ADG), FCR, and water intake (WI) according to the THI level of Hanwoo steers at the early-fattening period are reported in Table 2. Lower DMI of formula feed in Severe (4.88) was observed compared to Mild (5.11) and Comfort (5.05) (p<0.05), while Moderate (5.01 kg/d) had intermediate level. The DMI of rice straw was the lowest in the Severe (0.43) and lower in Moderate (0.46) than Comfort (0.50) and Mild (0.49 kg/d) (p<0.05). As a result, whole DMI was approximately 20% lower in Severe (4.31) than in Comfort (5.55) and Mild (5.61 kg/d).

One of the most obvious outcomes of heat stress was a decrease in DMI. Feed intake of beef cattle begins to decrease from ambient temperature of 26°C to 27°C, and decreases rapidly at temperature above 30°C [10]. Approximately 12% of reduced DMI in ruminants occurred within a range of ambient temperature between 29.4°C and 40°C [24]. These heat-stressed ruminants reduced diurnal activity, rumination, and energy metabolism to decrease production of metabolic heat by reducing feed intake. The reduction in feed intake due to heat stress has traditionally been considered a primary response and a means to decrease heat production in ruminants because rumination increases heat production [25]. Furthermore, heat stress has a direct negative impact on the appetite center in the hypothalamus, leading to decreased feed intake and reduced rumination time [26,27].

Heat stress leads to more reduce forage intake than formula feed intake. This is speculated to be due to the higher heat generated by microbial fermentation in forage compared to formula feed in the rumen-reticulum [22]. The results of this study was confirmed that both formula feed and roughage intake did not decrease in Comfort and Mild. However, formula feed intake did not decrease, whereas roughage intake significantly decreased in Moderate compared to Comfort and Mild. Both formula feed and roughage intake decreased in Severe. Therefore, based on this study, it can be seen that in extremely high THI conditions, especially in Severe, feed intake was reduced by up to 20%.

The BW gain of Comfort and Mild were 7.72 and 6.75, while those of Moderate and Severe decreased to 2.75, and 1.88 kg, respectively (p<0.05). When comparing the Comfort with Severe, there was a decrease of 76% in BW gain (p<0.05). The ADG showed a similar trend to the BW gain, being much lower at both Severe (0.19) and Moderate (0.28) than Mild (0.68) and Comfort (0.77 kg/d) (p<0.05). No significant difference was observed between Comfort and Mild, but numerical decreases were observed between these treatments. Thus, the THI level of Moderate or higher could have a significant negative effect on productivity. However, Moderate and Severe showed a decrease of 63% and 75% in ADG compared to Comfort, respectively. According to NIAS [19], Hanwoo steers during the fattening period under heat stress with THI levels of 77 to 79 and 81 to 83 resulted in decreased ADG due to increased respiratory rate, elevated body temperature, and decreased feed intake. Simultaneously, these conditions lead to reduce ruminal motility and increase feed retention time in the digestive tract, ultimately causing an additional energy expenditure by 10% of maintenance requirements [28]. Therefore, this may be the reason for the greater extent of reduction of ADG compared to DMI.

Statistical differences were noted for FCR in Severe (28.78) and Moderate (23.24) compared to Mild (9.94) and Comfort (7.40) (p<0.05). The FCR of Severe showed approximately 74.3% higher than Comfort. The FCR in Hanwoo steer during early-fattening period began to rise more rapidly at Moderate. The results of this study showed a clear difference in FCR with Moderate being about 57.2% higher than Mild (p<0.05). However, there was no significant difference between Moderate and Severe. This is probably because the THI levels of Moderate and Severe were 81 to 83 and 84 to 86, respectively, having relatively small differences. The influence of heat stress on beef cattle in terms of high THI can have a more negative effect on growth performance at the Moderate and Severe compared to the Comfort and Mild. These results implied that although the absolute value of each THI stage is not very large, the physiological responses and the subsequent effect on productivity become very large. In particular, the Moderate stage has very narrow THI value range (82 to 84), and at this stage or higher, livestock can undergo high-temperature stress.

The WI was the highest in Severe (37.66) and followed by Moderate (36.41) compared with Comfort (34.52) and Mild (34.85 L/d) (p<0.05). The WI of Hanwoo calves in climatic controlled chambers was 28% higher at THI 83 and 84 than at THI 74 [11]. The enhanced WI was intimately associated with increased THI for maintaining the normal range of body temperature [29,30]. Ruminants exposed to high temperature and humidity regulate their body temperature by increasing body sweat and panting of mouth. These water losses from the respiratory tract and thermoregulation can natively lead to an increase in WI, and this increase in WI can affect a decrease in DMI [10]. The dilution of rumen contents resulting from increased WI, decreased activity of rumen bacteria, reduced rumen motility, and diminished saliva production may potentially contribute to changes in digestibility when animals are chronically exposed to intense THI. It was proposed that ruminants exposed to heat exhibit lower feed digestibility, accompanied by decreased pH levels and concentrations of cellulolytic and amylolytic bacteria, slower passage of digesta, and lower osmolarity of rumen contents, indicating potential impairment of bacterial activity and significant rumen fluid dilution [31]. The negative impact of reduced rumen bacteria activity on feed digestibility may outweigh the positive effects resulting from decreased DMI and digesta outflow rate, thereby leading to a net decrease in feed digestibility in heat-stressed ruminants.

### Physiological parameters and blood profiles

The physiological and blood parameters according to the THI level on the early-fattening period of Hanwoo steers are presented in Table 3. The HR of Comfort, Mild, Moderate, and Severe increased in the order of 63.50, 69.00, 76.25, and 79.56 bpm, respectively (p<0.05). The RT also increased as the heat stress level increased (p<0.05). When comparing RT between Comfort (37.39°C) and Severe (39.20°C) THI conditions, there was a difference of 1.81°C, which corresponds to an approximate 4.6% increase. Homeothermic animals regulate their core body temperature through various physiological mechanisms, including dissipating heat from the body by adjusting their HR and respiratory rate, in order to maintain thermoregulatory stability [32].

According to the heat stress indicators of THI chart for Hanwoo steers proposed by NIAS [18], the HR and RT for the Mild (THI 76 to 81) level were in the range of 66 to 68 bpm and 38.5°C to 38.8°C, respectively. For the Moderate (THI 82 to 84) level, they were in the range of 69 to 75 bpm and 38.9°C to 39.2°C, respectively. It was also reported that the HR was above 75 bpm and the RT was above 39.3°C for the Severe (THI 85 to 99). When comparing the results of this study with those of NIAS [18], it was observed that both HR and RT corresponded to their respective THI stages. However, the HR of Moderate in this study was recorded as 76.2 bpm, which was above 75 bpm, and the RT showed 38.65°C. This was due to the fact that the THI range for Moderate was 82 to 84, which was very close to the THI range for Severe (THI 85 or above). In addition, Kim et al [11] reported that the HR (R^2^ = 0.84) and RT (R^2^ = 0.62) in ruminants were positively associated with THI. Animals are generally assessed for their HR and RT levels across different environments, where heightened HR and RT values are indicative of significant heat stress [24].

Serum glucose was the lowest in Severe (59.15) and lower in Moderate (63.29) compared to Comfort (69.59) (p<0.05), having intermediate value of Mild (66.72 mg/dL) between Comfort and Moderate. There were no statistical significance in glucose levels between Moderate and Severe. This is because the numerical difference between these two THI stages was very small. The NEFA was the highest in Severe (303.6) and lower in Moderate (264.7) compared with Comfort (213.7) and Mild (234.0 μEq/L) (p<0.05). Several studies reported that as THI increased, glucose decreased but NEFA increased [11,14,16,17]. Particularly, serum glucose concentrations of Hanwoo calves in climatic controlled chambers were decreased at THI of 83 (56.8) compared to that of 70 (73.5) and 77 (72.5 mg/dL), whereas NEFA concentrations were increased at THI from 84 (303.7) and 88 (288.3) compared to that at THI of 70 (126.3 μEq/L) [11]. These metabolite changes such as decreased glucose and increased NEFA concentrations under heat stress could be attributed to variations in feed intake, because the experimental animals were fed the same diets containing equal amounts of energy and protein [14,24]. Metabolite changes may be caused by decreased energy intake due to decreased feed intake as a result of heat stress, increased energy expenditure for thermoregulation, and negative effects on gluconeogenesis, which could be an endocrine adaptation to cope with a hot environment [33].

Substantial alterations in both carbohydrate and lipid metabolism occur to ensure the prioritization of nutrients from both dietary sources and tissues towards muscle. These changes are facilitated by variations in the levels of anabolic and catabolic signals, which play a pivotal role in regulating these metabolic shifts [34]. One of these features is that lower blood glucose levels can lead to decreased insulin secretion or increased insulin resistance. This results in an increase in the breakdown and conversion of fatty acids in adipose tissue to NEFA for using an energy source in the body [24].

The BUN of Moderate (13.71) and Severe (14.39) showed differences from both Comfort (12.55) and Mild (12.75 mg/dL) (p<0.05). The increase in BUN concentrations with the increase of THI remained uncertain whether it was attributed to skeletal muscle breakdown or excessive ammonia production due to heat stress [13,35]. Otherwise, it may be due to an imbalance in protein metabolism due to a lack of energy.

Serum cortisol of Comfort, Mild, Moderate, and Severe were 11.96, 13.51, 15.57, and 17.17 ng/mL (p<0.05). Cortisol concentrations of Hanwoo calves in climatic controlled chambers were also higher at THI of 84 (15.9) and 88 (17.1) than that at 70 (4.8 ng/mL) [11]. Another research result [35] of Hanwoo steers in outside environments was that increased blood cortisol levels were observed after exposure to THI 80 to 87 (9.87) compared to THI 64 to 71 (1.91) and THI 72 to 79 (5.13 ng/mL). In addition, NIAS [18] reported that levels of blood cortisol in fattening stage of Hanwoo stress were 1 to 4 ng/mL in Mild, 5 to 6 ng/mL in Moderate and more than 6 ng/mL in Severe. In the results of this study, the cortisol concentration was 11.98 ng/mL in Comfort, which was much higher compared to the level reported by NIAS [18]. This difference may be due to the stress of the animals during the chamber experiment. However, in this experiment, cortisol levels increased to a much greater extent with increasing THI. Generally, increases in cortisol have been closely associated with abnormal behaviors such as anxiety and hyper-sensitivity in animals [36]. These factors could potentially have a detrimental impact on the productivity of livestock, as it may adversely affect their overall body condition [37]. Overall, RT, HR, serum glucose, and cortisol increased with increasing levels of THI, while NEFA decreased. Based on these physiological and blood analytical results of this study, it is judged that the THI chart for Hanwoo steers of NIAS [18] was properly calculated.

## CONCLUSION

This study investigated the effects of heat stress on growth performance, physiological parameters, and blood profiles of Hanwoo steers during their early-fattening period. The results revealed significant changes in performance and physiological parameters as THI increased. In particular, this study found that BW gain and DMI decreased as HS increased from Mild to Severe. Physiological responses to heat stress such as increased HR and RT demonstrated the efforts of the steers to cope with rising THI. Additionally, heat stress led to alterations in blood profiles, with decreased serum glucose and increased NEFA. Based on these results, the THI chart for Hanwoo steers from NIAS [18] appears to be appropriately calculated.

Heat stress significantly affected growth performance of the Hanwoo steers in both Moderate and Severe conditions. Feed intake decreased as THI levels rose, resulting in reduced ADG. Severe heat stress also caused the steers to increase WI. Overall, this study demonstrated that the productivity decrease due to heat stress in early-fattening period of Hanwoo steers was more likely to occur above Moderate conditions. By precisely measuring metabolic changes in Hanwoo steers of early-fattening period and their relationship to the environment, these data could be used to prepare specific feeding management for the summer season and to determine standards for animal welfare.

## Figures and Tables

**Table 1:** Ingredients of formula feed and chemical composition of experimental diets in Hanwoo steers of early-fattening period

Items (%)	Formula feed	Rice straw
Ingredients (as-fed)		
Corn flake	40.06	
Wheat grain	1.80	
Molasses	5.00	
Corn gluten feed	25.10	
Soybean meal	1.20	
Rapeseed meal	2.50	
Canola meal	3.40	
Wheat bran	7.00	
Corn distiller’s dried grains with solubles	3.92	
Palm kernel meal	6.60	
Limestone	2.10	
Salt	0.60	
Vitamin and mineral premix	0.72	
Total	100	
Chemical composition (DM)		
Dry matter	86.83	85.71
Crude protein	14.51	4.22
Ether extract	3.78	1.04
Ash	5.34	9.85
Neutral detergent-insoluble fiber	36.79	76.35
Acid detergent-insoluble fiber	14.96	49.13
Acid detergent lignin	4.68	8.72
Total digestible nutrients	74.87	52.48

**Table 2:** Effects of level of heat stress on growth performance in Hanwoo steers of early-fattening period

Item	THI^1)^				SEM	p-value

	Comfort	Mild	Moderate	Severe		
Dry matter intake (kg/d)						
Formula feed	5.05^a^	5.11^a^	5.01^ab^	4.88^b^	0.108	0.039
Rice straw	0.50^a^	0.49^a^	0.46^b^	0.43^c^	0.013	<0.001
Whole	5.55^a^	5.61^a^	5.47^ab^	4.31^b^	0.101	0.019
Body weight gain (kg)	7.72^a^	6.75^a^	2.75^b^	1.88^b^	0.871	<0.001
Average daily gain (kg/d)	0.77^a^	0.68^a^	0.28^b^	0.19^b^	0.087	<0.001
Feed conversion ratio (F/G)	7.40^b^	9.94^b^	23.24^a^	28.78^a^	2.803	<0.001
Water intake (L/d)	34.52^c^	34.85^c^	36.41^b^	37.66^a^	0.462	<0.001

SEM, standard error of the means.

1) THI, temperature-humidity index; Comfort, THI 73 to 75; Mild, THI 77 to 79; Moderate, THI 82 to 84; Severe, 85 to 86.

a–c Means within a row without a common superscript letter differ (p<0.05).

**Table 3:** Effects of level of heat stress on physiology parameters and blood profiles in Hanwoo steers of early-fattening period

Item	THI^1)^				SEM	p-value

	Comfort	Mild	Moderate	Severe		
Heart rate (bpm)	63.50^d^	69.00^c^	76.25^b^	79.56^a^	0.425	<0.001
Rectal temperature (°C)	37.39^d^	37.80^c^	38.65^b^	39.20^a^	0.087	<0.001
Glucose (mg/dL)	69.59^a^	66.72^ab^	63.29^bc^	59.15^c^	3.639	<0.001
TG (mg/dL)	14.20	14.92	15.56	14.17	0.619	0.259
Albumin (g/dL)	3.37	3.40	3.35	3.43	0.060	0.759
BUN (mg/dL)	12.55^b^	12.75^b^	13.71^a^	14.39^a^	0.840	<0.001
Total protein (g/dL)	7.03	6.96	7.28	7.15	0.161	0.418
HDL (mg/dL)	82.22	83.08	79.85	80.33	2.268	0.359
Cholesterol (mg/dL)	142.7	146.1	142.9	145.5	3.778	0.882
NEFA (μEq/L)	213.7^c^	234.0^c^	264.7^b^	303.6^a^	25.90	<0.001
Cortisol (ng/mL)	11.98^d^	13.51^c^	15.57^b^	17.17^a^	1.182	<0.001
WBC (k/μL)	11.23	10.85	10.38	11.01	0.479	0.556
Lymphocyte (k/μL)	6.02	6.37	5.35	6.05	0.399	0.277
Platelet (k/μL)	360.7	385.0	378.6	376.9	14.80	0.638

SEM, standard error of the means.; bpm, beat per minute; TG, triglyceride; BUN, blood urea nitrogen; HDL, high-density lipoprotein; NEFA, non-esterified fatty acid; WBC, white blood cell.

1) THI, temperature-humidity index; Comfort, THI 73 to 75; Mild, THI 77 to 79; Moderate, THI 82 to 84; Severe, 85 to 86.

a–d Means within a row without a common superscript letter differ (p<0.05).
